# Analysis of factors associated with the first lumpy skin disease outbreaks in naïve cattle herds in different regions of Thailand

**DOI:** 10.3389/fvets.2024.1338713

**Published:** 2024-02-22

**Authors:** Orapun Arjkumpa, Wanwisa Wachoom, Bopit Puyati, Sirima Jindajang, Minta Suwannaboon, Sith Premashthira, Tippawon Prarakamawongsa, Tosapol Dejyong, Chalutwan Sansamur, Roderick Salvador, Chalita Jainonthee, Veerasak Punyapornwithaya

**Affiliations:** ^1^Animal Health Section, The 4th Regional Livestock Office, Department of Livestock Development, Khon Kaen, Thailand; ^2^Nawa District Livestock Office, Department of Livestock Development, Nakhon Phanom, Thailand; ^3^Buriram Provincial Livestock Office, Department of Livestock Development, Buriram, Thailand; ^4^Animal Health Section, The 7th Regional Livestock Office, Department of Livestock Development, Nakhon Pathom, Thailand; ^5^Regional Field Epidemiology Training Program for Veterinarian, Bureau of Disease Control and Veterinary Services, Department of Livestock Development, Bangkok, Thailand; ^6^Bureau of Disease Control and Veterinary Services, Department of Livestock Development, Bangkok, Thailand; ^7^Akkhararatchakumari Veterinary College, Walailak University, Nakhon Si Thammarat, Thailand; ^8^College of Veterinary Science and Medicine, Central Luzon State University, Science City of Muñoz, Philippines; ^9^Veterinary Public Health and Food Safety Centre for Asia Pacific (VPHCAP), Faculty of Veterinary Medicine, Chiang Mai University, Chiang Mai, Thailand; ^10^Research Center for Veterinary Biosciences and Veterinary Public Health, Faculty of Veterinary Medicine, Chiang Mai University, Chiang Mai, Thailand

**Keywords:** lumpy skin disease, risk factors, cattle herds, control measures, Thailand

## Abstract

**Introduction:**

Thailand experienced a nationwide outbreak of lumpy skin disease (LSD) in 2021, highlighting the need for effective prevention and control strategies. This study aimed to identify herd-level risk factors associated with LSD outbreaks in beef cattle herds across different regions of Thailand.

**Methods:**

A case–control study was conducted in upper northeastern, northeastern, and central regions, where face-to-face interviews were conducted with farmers using a semi-structured questionnaire. Univariable and multivariable mixed effect logistic regression analyses were employed to determine the factors associated with LSD outbreaks. A total of 489 beef herds, including 161 LSD outbreak herds and 328 non-LSD herds, were investigated.

**Results and discussion:**

Results showed that 66% of farmers have operated beef herds for more than five years. There were very few animal movements during the outbreak period. None of the cattle had been vaccinated with LSD vaccines. Insects that have the potential to act as vectors for LSD were observed in all herds. Thirty-four percent of farmers have implemented insect control measures. The final mixed effect logistic regression model identified herds operating for more than five years (odds ratio [OR]: 1.62, 95% confidence interval [CI]: 1.04–2.53) and the absence of insect control management on the herd (OR: 2.05, 95% CI: 1.29–3.25) to be associated with LSD outbreaks. The implementation of insect-vector control measures in areas at risk of LSD, especially for herds without vaccination against the disease, should be emphasized. This study provides the first report on risk factors for LSD outbreaks in naïve cattle herds in Thailand and offers useful information for the development of LSD prevention and control programs within the country’s context.

## 1 Introduction

Lumpy skin disease (LSD) is a highly contagious viral disease that primarily affects cattle. It is caused by the lumpy skin disease virus (LSDV), a member of the *Capripoxvirus* genus (1). Clinical manifestations of LSD can include fever, loss of appetite, and general weakness. The most notable feature, however, is the appearance of the characteristic skin nodules. These nodules can occur on various parts of the body, including the head, neck, limbs, and genital areas (2). In severe cases, the nodules may become ulcerated, leading to secondary bacterial infection. LSD poses significant economic implications for cattle populations. In affected herds, the morbidity rate can vary widely, ranging from 3 to 85%, depending on the susceptibility of cattle and other factors (3, 4). The mortality rate is typically lower than 3% (3), but in some cases, it may exceed 40% (5). The disease can have devastating effects on the livestock industry, leading to substantial economic losses. The World Organization for Animal Health (WOAH) has defined LSD as a disease requiring notification due to the potential for rapid virus propagation in susceptible cattle populations and the consequential considerable economic effects in affected herds (6, 7).

While LSD was previously confined to Africa with occasional incursions into the Middle East, recent outbreaks have raised concerns about its emergence and rapid spread in Asia (4, 8–14). Thailand, being a significant hub for livestock production and trade in Southeast Asia, has also experienced the impact of LSD outbreaks since March 2021 (15). It was initially detected in the cattle farming regions located in the northeastern part of Thailand (9). Later, outbreaks of LSD were reported across the country. There were 283,213 affected herds with 628,089 cases across 64 provinces as of June 30, 2022 (12).

Various risk factors associated with LSD outbreaks in endemic settings have been identified (16, 17). The movement of infected animals is considered as a significant factor in facilitating long-range transmission, whereas arthropod-borne transmission is likely to be the primary mechanism responsible for the rapid and aggressive spread of the disease over short distances (18). The predominant blood-feeding arthropod vectors for LSD are stable flies (*Stomoxys calcitrans*), mosquitoes (*Aedes aegypti*), and hard ticks (*Rhipicephalus* and *Amblyomma* species) (1). Furthermore, cattle breed, source of replacement stock, introduction of new animals, herd size, communal grazing and watering management, and housing were identified as potential risk factors for the LSD outbreak in previous studies (16, 19–22). Moreover, management type, gender, age, precipitation, and intake of community water sources have been determined to be risk factors for LSD (23). However, there is a notable research gap regarding the specific risk factors for LSD in the context of Thailand.

Understanding the risk factors associated with the occurrence of LSD is crucial for effective prevention and control strategies. Identifying and quantifying these factors can aid in the development of targeted interventions, including vaccination campaigns, vector control measures, and improved biosecurity practices. Therefore, this study aims to determine the risk factors contributing to the occurrence of LSD outbreaks in naïve cattle herds in various regions of Thailand. The finding from this study has the potential to significantly advance the development of targeted control measures and policies. Ultimately, this will lead to improved management and prevention of the disease. The outcomes of this study may also contribute to the existing body of knowledge on LSD risk factors, potentially benefiting other countries facing similar challenges.

## 2 Materials and methods

### 2.1 Study population and sampling

**Figure 1:**
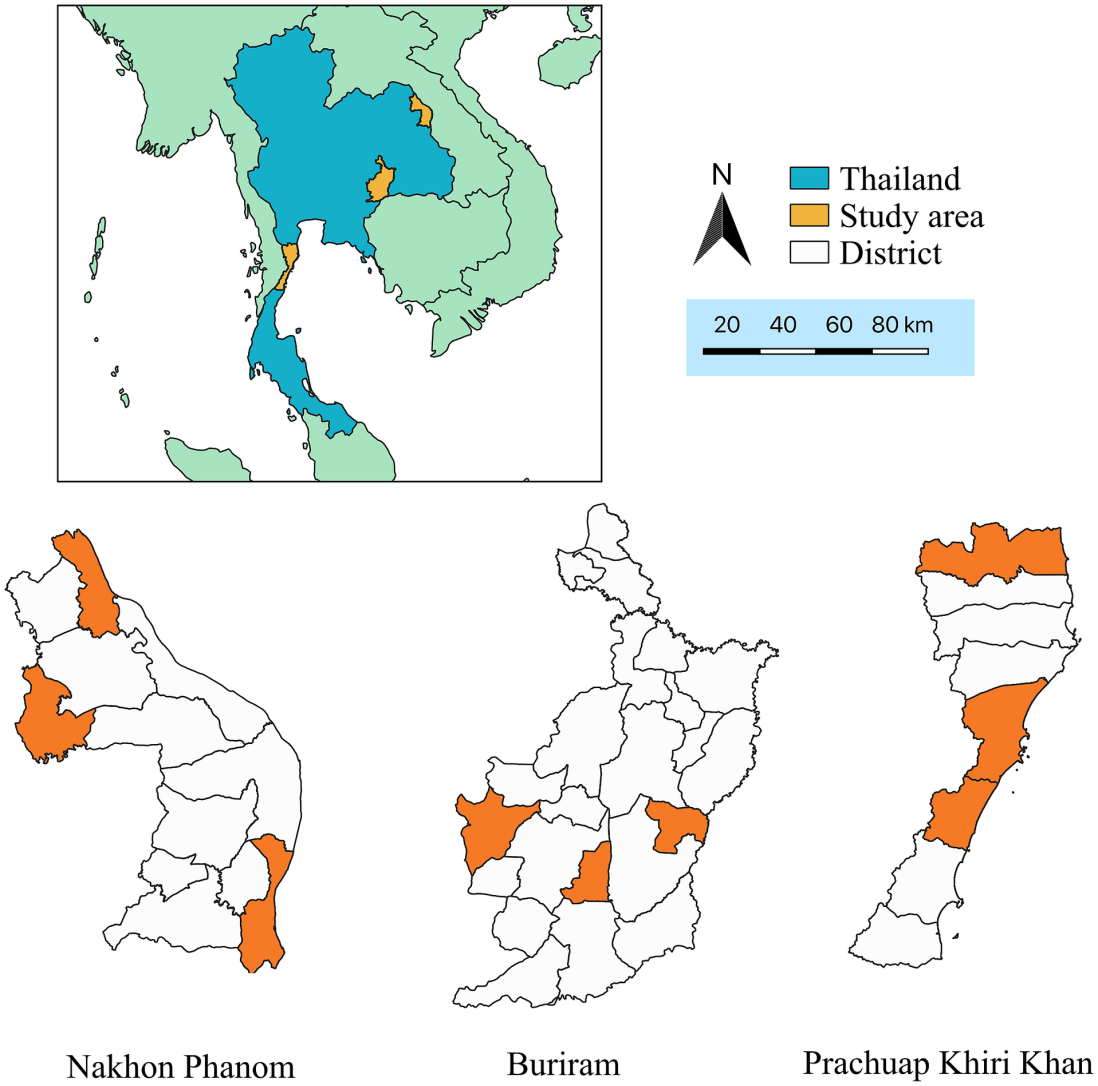
Map of Thailand displaying study areas (orange color) which are located in Nakhon Phanom, Buriram, and Prachuap Khiri Khan provinces.

This case–control study was conducted in three provinces of Thailand: Nakhon Phanom, Buriram, and Prachuap Khiri Khan (Figure 1). The study took place from July to September in Nakhon Phanom, and from August to September in both Buriram and Prachuap Khiri Khan, all in the year 2021. It is important to note that the questionnaire survey was not conducted during the outbreak period, as the primary investigation prioritized the outbreak investigation protocol carried out by livestock authorities in each area. It is noteworthy that the surveys in all three provinces were carried out approximately 2 months after the latest herd had confirmed the LSD outbreak. Furthermore, the study focused on households that owned cattle as the primary unit of analysis. To ensure representative samples, a multi-stage sampling technique was employed.

Initially, the selection of provinces was purposive and based on collaboration between central and local veterinary authorities. Subsequently, within each province, three districts were chosen using a simple random sampling approach. Furthermore, subdistricts within each district were randomly selected. The case herds in this study were identified based on the official outbreak investigation reports issued by local veterinary authorities in each subdistrict. In each sub-district area, all LSD outbreak herds were included in the study. Control herds were randomly selected from herds located in the same sub-village as the case herds. An approximately 1:2 ratio for case and control herds, respectively was applied. As a result, the total number of herds included in this study for Nakhon Phanom, Buriram, and Prachuap Khiri Khan provinces was 159, 180, and 150, respectively.

### 2.2 Case and control definitions

Cattle herd served as the epidemiological unit. A case herd, or an LSD-outbreak herd, was defined as a herd with at least one individual cattle showing the LSD clinical signs, which include raised, circular, firm, nodules varying from 1 to 7 cm diameter, as observed by investigators from the Department of Livestock Development (DLD) (9). Confirmation of the disease could be through laboratory testing using the polymerase chain reaction (PCR) method (12), although it was not always a prerequisite. A control herd, or a non-LSD outbreak herd, was defined as a beef cattle herd located in the same village and/or subdistrict as the case herds. The control herds must not have any history of clinical LSD among their animals. The historical records of LSD outbreaks were cross-checked with information provided by farmers and local veterinary authorities during the questionnaire survey.

### 2.3 Questionnaire survey

The semi-structured questionnaire utilized in this study was developed collaboratively by veterinary experts from the DLD and epidemiologists from the Regional Field Epidemiology Training Program for Veterinarians (R-FETPV), supported by the Food and Agriculture Organization of the United Nations (FAO). Several questions in the questionnaire were adopted from the official outbreak investigation form employed for nationwide investigations of LSD outbreaks. The questionnaire covered various relevant variables including the owner’s profile, farm characteristics, biosecurity, and other management practices.

Data collection was carried out by livestock and veterinary authorities. In cases where data were incomplete, follow-up telephone interviews were conducted to gather the necessary information.

### 2.4 Hierarchical structure of the data

The data is organized into a hierarchical structure, wherein it is structured into multiple levels or layers, with each level representing distinct units of study. Within the study’s dataset, farms are grouped into clusters within districts, and these districts, in turn, are clustered within provinces. This hierarchical arrangement facilitates statistical analyses.

### 2.5 Statistical analysis

#### 2.5.1 Descriptive analysis

Descriptive statistics, including the mean and standard deviation for quantitative variables, as well as frequencies (expressed as percentages) for qualitative variables, were calculated using R version 3.6.2 (https://www.r-project.org).

#### 2.5.2 Univariable mixed effect logistic regression analysis

The mixed effect univariable logistic regression model used in this study incorporated both fixed and random effects. Each potential risk factor is defined as a fixed effect, while the individual district was included as a random effect (24). To account for the clustering of districts within provinces, a factor named “province” was included in the univariable and multivariable logistic models as a fixed effect (25). The odds ratio and *p*-value were determined based on Wald’s test.

Subsequently, risk factors with a *p*-value less than 0.2 were selected for further analysis using a mixed effect multivariable logistic regression. The objective of this step was to select factors that have a significant association with the outcome while accounting for potential confounding variables. Multicollinearity between variables was also examined using a Cramer’s π-prime statistics. A pair of categorical variables was considered collinear if Cramer’s π-prime statistics was greater than 0.7 (24, 26).

#### 2.5.3 Multivariable mixed effect logistic regression analysis

##### 2.5.3.1 Model

In the mixed effect multivariable logistic regression model, the potential risk factors were considered as fixed effects, while the individual district was defined as a random effect, similar to a previous study (21). The models also incorporated the variable “province” as a fixed effect as suggested in the literature (25). The statistical model can be expressed as follows (27):


$$\mathrm{logit}\left[\mathrm{Pr}\left(y_{ij}=1\right)\right]=\beta_{0}+\beta_{1}x_{1i}+..+\beta_{k}x_{ki}+u_{\mathrm{district}\left(i\right)}+\varepsilon_{i}$$

where $y_{ij}$ is the outbreak status (1 = outbreak or 0 = non-outbreak) of a herd i clustered in district j . The term $\beta_{0}$ represents the intercept, $\beta_{k}$ is the regression coefficient for the fixed effect factors $\left(\beta_{k}=1,..,k\right)$ and $x_{k}$ is a set of fixed effect factors $\left(x_{k}=1,..,k\right)$ . The term $u_{\mathrm{district}\left(i\right)}$ is the random effects on the intercept for the j district which includes herd i . It was assumed that $u_{\mathrm{district}\left(i\right)}∼N\left(0,\sigma^{2}\right)$ . The error terms $\varepsilon_{i}$ are assumed to follow a logistic distribution with mean zero and variance $\pi^{2}/3$ .

##### 2.5.3.2 Model selections

Model selection was performed using a backward stepwise method. Akaike’s Information Criteria (AIC) was utilized as the criterion for selecting the most appropriate model (23, 28–31). The interaction between variables was also examined during the model selection process. If the inclusion of an interaction term did not improve the model, the interaction term was removed from the model.

Confounding was assessed by examining the change in estimated coefficients of the variables that remained in the final model upon the addition of a non-selected variable. If the inclusion of this new variable resulted in a change of >25% in any parameter estimate, that variable was deemed a confounder and retained in the model (24, 26).

##### 2.5.3.3 Evaluation of multicollinearity and model assumptions

After identifying the final model, an assessment of multicollinearity was conducted by examining the variance inflation factors (VIF) values. The VIF represents the ratio of the overall variance in the model to the variance when a specific single variable is included. A VIF value below 5 indicates no evidence of multicollinearity among the variables included in the final model (32). Additionally, residual diagnostics for the final mixed effect model were evaluated.

##### 2.5.3.4 In the final model, odds ratios and their corresponding 95% confidence intervals were calculated for each variable intra-class correlations

For the final model, we considered the variance components as a random effect, dividing them into two levels based on their origin. The first level variance is equivalent to $\frac{\pi^{2}}{3}$ on the logit scale, and this represents the error variance in the binary model. The second level variance symbolizes the random intercept that changes based on the district’s effect, symbolized as $\sigma_{j}^{2}$ . As a result, to illustrate these variances, we calculated the intra-class correlation (ICC). The formula used for calculating the ICC is as follows (25):


$$ICC=\frac{\sigma_{j}^{2}}{\sigma_{j}^{2}+\frac{\pi^{2}}{3}}$$

A low ICC indicates minimal clustering as most of variance is found within individual districts. In contrast, a high ICC means that there is less variation within a district when compared to the variation observed between the different districts (33).

The mixed effect logistic regression was conducted using the “glmer” function from the “lme4” package. To assess the variance inflation factors (VIF), the “vif” function from the “car” package was employed. The diagnostics of residuals were carried out using “DHAMa” package. The ICC was obtained from “mlmhelpr” package.

## 3 Results

### 3.1 Respondent and management practices

A total of 161 LSD-outbreak herds and 328 non-LSD outbreak herds from three provinces in Thailand participated in this study. The provinces included Buriram (*n* = 180), Nakhon Phanom (*n* = 159), and Prachuap Khiri Khan (*n* = 150). The average age of the participants was 54 in the case group and 53 in the control group. Males constituted approximately 72% of the respondents in both groups (Table 1). Most respondents in both groups had a primary education. The average duration of herd operation was 7.7 years with a median of 5 years, The average number of cattle per herd was 4.6 with a median of 5 animals. The majority of herds (90%) had facilities for keeping cattle in stalls.

**Table 1:** Characteristics of respondents, and LSD case (herd with LSD outbreak) and control (herd without LSD outbreak) herds enrolled in a case–control study of risk factors associated with lumpy skin disease outbreaks in beef herds in Thailand.

Variables	Categories	LSD case herds (*n* = 161)		LSD control herds (*n* = 328)	
		*N* (%)	Mean (SD)	*N* (%)	Mean (SD)
**Respondents**					
Provinces					
	Buriram	60 (37.27)		120 (36.59)	
	Nakhon Phanom	51 (31.68)		108 (32.93)	
	Prachuap Khiri Khan	50 (31.06)		100 (30.49)	
Gender					
	Male	116 (72.05)		236 (71.95)	
	Female	45 (27.95)		92 (28.05)	
Age (year)			53.66 (11.99)		52.60 (12.04)
Education level					
	Primary level	93 (57.76)		206 (62.80)	
	Secondary level	56 (34.78)		85 (25.91)	
	Other	12 (7.46)		91 (11.29)	
**Beef cattle herds**					
Farming operation (year)			8.32 (5.76)		6.65 (5.53)
Beef cattle population (heads)			4.3 (5.83)		4.8 (5.91)

Farm characteristics and management practices for the herds included in this study are summarized in Table 1. Out of all the herds investigated, only eleven herds had a history of purchasing cattle from other herds and transporting them to their own facilities. All herds examined reported the presence of stable flies or mosquitoes or both. Notably, none of the herds had a history of using LSD vaccines. Additionally, the data highlights that 36% of herds with an operational history exceeding 5 years experienced LSD outbreaks, while the percentage was lower at 25% for herds operated for 5 years or less (Table 2). Insect control measures have been adopted by 34% of farmers. Among those who did not implement these measures, 40% experienced an LSD outbreak, while only 28% of farmers who employed such control measures encountered outbreaks (Table 2). Risk factors associated with LSD outbreaks.

**Table 2:** Summary of associated risk factors related to lumpy skin disease in cattle of herd level based on univariable logistic regression analysis in 3 provinces (*n* = 489).

Variables	Categories	Case	Control	OR (95%CI)	*p*-value
Years in operation	>5	120	209	1.6 (1.03–2.48)	0.04
	≤5	41	119	--Ref*--	
Herd size	>5	69	164	1.28 (0.85–1.93)	0.23
	≤5	92	164	--Ref--	
Having a calf age less than a year	Yes	103	186	1.27 (0.83–1.95)	0.27
	No	58	142	--Ref--	
Having other animals on the herd	Yes	49	123	0.67 (0.41–1.10)	0.11
	No	112	205	--Ref--	
Using a communal water source	Yes	61	115	1.36 (0.84–2.20)	0.21
	No	100	213	--Ref--	
Raise cattle by public grass grazing	Yes	36	78	0.97 (0.58–1.62)	0.92
	No	125	250	--Ref--	
Absence of biosecurity fencing	Yes	85	155	1.28 (0.79–2.08)	0.31
	No	76	173	--Ref--	
Absence of disinfectants	Yes	123	226	1.27 (0.80–2.01)	0.32
	No	38	102	--Ref--	
Lacking restriction control for vehicle that visit the herd	Yes	87	184	0.77 (0.47–1.27)	0.31
	No	74	144	--Ref--	
Contact of cattle with other herds	Yes	28	48	1.27 (0.73–2.20)	0.40
	No	133	280	--Ref--	
Lacking manure removal from the farm	Yes	87	185	0.98 (0.58–1.65)	0.93
	No	74	143	--Ref--	
Introduction of new cattle during the last 2 months	Yes	3	8	0.77 (0.19–3.22)	0.73
	No	158	320	--Ref--	
Presence of at least one type of insect including stable flies and mosquitoes the farm	Yes	161	328	NA	NA
	No	0	0	--Ref--	
Lacking vector management practice	Yes	68	99	2.44 (1.55–3.83)	<0.001
	No	93	229	--Ref--	

*Ref = reference category.

### 3.2 Risk factors

The risk factors for LSD outbreaks identified in this investigation, as determined by univariable logistic regression, are presented in Table 2. The analysis revealed that the number of years in operation and the absence of vector management on the herd were associated with the LSD outbreak status.

In the final multivariable mixed effect logistic regression model (Table 3), results showed that cattle herds operating for more than five years had 1.62 times greater odds of experiencing an LSD outbreak (OR = 1.62; 95%CI = 1.04–2.53) than those operating for fewer years. Furthermore, herds that did not implement insect vector control measures had 2.05 times greater odds of being affected by LSDV (OR = 2.05; 95%CI = 1.29–3.25) compared to those implementing these control measures.

**Table 3:** Risk factors from the final multivariable logistic regression model* for the lumpy skin disease outbreak in cattle herds at the herd level.

Variables	Categories	Estimate	Standard error	Adjusted odd ratio (95%CI)	*p*-value
(Intercept)		−1.19	0.41	0.30 (0.14–0.38)	0.004
Province	NKP PKK BRR	−0.11 −0.63	0.54 0.58	0.90 (0.31–2.59) 0.53 (0.17–1.66) --Ref**--	0.84 0.27
Year in operation	>5 ≤ 5	0.48	0.23	1.62 (1.04–2.53) --Ref--	0.032
Lacking vector management practice	Yes No	0.72	0.24	2.05 (1.29–3.25) --Ref--	0.002

Hosmer-Lemeshow test (*p* = 0.07), Akaike information criteria (AIC) = 609.72, NKP = Nakhon Phanom, PKK = Prachuap Khiri Khan, BRR = Buriram. **Ref = reference category.

During the model selection step, no significant interaction term was identified in the final model. Furthermore, there was no evidence of multicollinearity among the variables included in the final model, as all variables included in the final model had VIF values of less than 1.04. The ICC from the final model was equal to 0.09, indicating that the effects of the variation observed within the district were smaller compared to the variation between the different districts.

Results related to the residual diagnostics for the final mixed effect model, including QQ plot residuals and a plot between residuals and predicted values, are displayed in the Supplementary Figure S1. The results demonstrate a lack of violations in the model assumptions.

## 4 Discussion

This study aimed to identify the risk factors associated with LSD outbreaks in naïve beef cattle herds located in the upper northeast, northeast, and central regions of Thailand. This research is an integral component of a national project that seeks to comprehend the epidemiology of LSDV, which has caused a significant outbreak in the country. The findings from this study hold the potential to contribute valuable insights to the national strategy for disease prevention and control.

Blood-sucking insects play a significant role in the mechanical transmission of LSDV (34–36). Various bloodsucking arthropods, such as mosquitoes (*Aedes aegypti*), stable flies (*Stomoxys calcitran*), horn flies (*Haematobia irritans*), house flies (*Musca domestica*), and hard ticks (*Dermacentor marginatus*, *Hyalomma asiaticum*, *Rhipicephalus appendiculatus*, *Rhipicephalus decoloratus*, and *Amblyomma hebraeum*), have been previously identified as potential transmitters of LSDV (37–39). Additionally, recent studies have confirmed that LSDV can be transmitted by insect vectors from animals infected with LSDV to animals that are susceptible to the disease (34, 40, 41). Based on mixed effect logistic regression analysis, this study determined that lack of vector control on the herds was identified as a significant risk factor for LSD outbreaks. In other words, herds of farmers who did not apply insect vectors control practices had 2.05 times greater odds for LSD outbreak than herds of farmers who did apply such practices. This finding provides support for the results from previous investigation conducted in other areas in Thailand (9), which reported that naïve cattle herd affected by LSD were primarily characterized by suboptimal insect control measures. Furthermore, all cattle herds in the present study were found to harbor insects that could potentially act as vectors for LSD. Thus, with inefficient insect vector control, it was revealed that the transmission of LSD in the naïve herds in this study is likely due to insect vectors. This speculation is supported by previous spatial epidemiological studies conducted in Thailand reporting that insect vectors play a crucial role in LSD outbreaks in cattle farming areas where herds are closely situated or in regions with a high concentration of cattle herds (9, 42, 43). In addition to the findings of the current study, a study conducted in Thailand, employing transmission kernel analysis, similarly affirms that herd-to-herd transmission in LSD outbreak areas occurs within short distances, with the estimated range falling between 0.2 and 0.8 kilometers (44). This discovery emphasizes the pivotal role that insects may play as significant vectors in the transmission among cattle herds. Furthermore, aligning with the outcomes of our study, the absence of insect vector control measures on farms emerges as a notable risk factor for LSD outbreaks in Indonesia. This investigation demonstrates that farms without insect vector control measures had 8.6 times (OR = 8.6) greater odds for experiencing an LSD outbreak compared to those implementing such measures (45). The impact of insect vectors on LSD transmission has been also observed in different settings. For example, in Sub-Saharan Africa, LSD outbreaks are typically observed following the rainy season when insect populations increase (46). A study conducted in Israel also demonstrated a correlation between the relative abundance of insect vectors in December and April and LSD outbreaks (47). Similarly, in various regions of Nepal, LSD outbreaks were reported during the rainy season (June to August), indicating a link to the increased population of arthropods in the area (48). Furthermore, in terms of implications, eliminating insects on a large scale is deemed impossible due to the common abundance of insect vectors in cattle farming areas throughout the year in Thailand (9). We recommend concentrating on measures to manage and mitigate the role of disease-transmitting vectors. This includes controlling breeding sites for insects, such as standing water and cattle manure. Additionally, the application of insecticides for vector control may be considered, but caution is advised, taking into account potential impacts on human health and the environment. The present study also showed that herds operating for more than five years had higher odds of experiencing LSD outbreaks compared to herds operating for less than five years. However, it is challenging to explain this finding. Although we examined the association between the total years of operation and other variables such as insect control and farm biosecurity, none of the pairs demonstrated a significant association. We hypothesize that farmers who possess over five years of experience may exhibit different farming practices in comparison to other groups of farmers. For example, individuals may exhibit a decreased propensity to obtain news or updates through online channels, which serve as a primary means of disseminating information regarding the LSD outbreaks in Thailand (12). To address this knowledge gap, a follow up study should be conducted to investigate this factor. Additionally, further investigation is necessary to investigate other risk factors that were not considered in this study.

Purchasing and selling animals during LSD outbreaks are determined as important risk factors of LSD outbreaks according to the study in Kazakhstan (21) and Indonesia (45). These factors were not identified as risk factors in this study. Strict animal movement restriction to mitigate LSD spread in Thailand was implemented during the course of this research. Only 2% of cattle herds included in this study have a history of animal movement limiting the evaluation of its impact to the occurrence of LSD. Another risk factor linked to the incidence of LSD was the size of the herd. Larger herds were found to have a higher risk of LSD infection, which can be attributed to factors such as stressful conditions, increased likelihood of exposure to the LSD virus, and greater possibilities for disease transmission (49). However, in this study, herd size was found to be less significant, mainly because most herds were small, typically consisting of around 5 cattle each, as they were predominantly owned by small-scale farmers.

Based on the findings of this study, it is recommended to implement insect control measures in LSD outbreak areas where no LSD vaccine is available, particularly for naïve herds. For herds that have been vaccinated against LSD, the use of insecticides can be an additional option, taking into account factors such as the abundance of insect vectors, the effectiveness of insecticide application, and economic considerations (7). It’s also important to point out that the source of the LSD outbreaks in the study areas was not determined. While the results suggest no correlation between LSD outbreaks and animal movements such as buying animals from other herds, it is crucial to remember that a small number of herds included in the study did have a history of animal movement. Thus, the sample size might not be large enough to fully examine the impact of this variable. In the study areas, we hypothesize that the origin of LSD outbreaks could be due to unauthorized movement of LSDV-infected cattle into the affected regions. Alternatively, the insects carrying the LSDV might have been introduced to the study areas either by flying or being transported by vehicles from other outbreak areas. Once an outbreak occurred, the spread of LSDV was likely aided by the high abundance of insect vectors in the outbreak regions, as suggested by previous studies (9, 12).

This study is subject to certain limitations. As it relied on a questionnaire survey, there is a possibility of recall bias and information bias, which are inherent to this type of study design. Furthermore, the presence of similar management factors in both outbreak and non-outbreak herds, as these practices were implemented in both types of herds, poses challenges in conducting statistical comparisons. Moreover, it should be noted that the diagnosis of LSD is primarily based on clinical signs, and as a result, subclinical cases may be included in the control group. However, given that most herds are naïve, cattle affected with LSDV would likely exhibit clinical signs of the disease (9). Therefore, the occurrence of subclinical cases in the control herd is less likely, but it should still be acknowledged as a limitation. Furthermore, it is important to note that the study was only conducted in a naïve herd. Therefore, interpretations of the results should take this condition into consideration.

Despite certain limitations, this study represents the first investigation of potential risk factors for LSD outbreaks in Thailand. The research was conducted across multiple sites throughout the country, providing a more comprehensive understanding compared to a study limited to a single area. Also, the sample size falls within the range of previously reported studies, being larger than some conducted to determine risk factors for LSD (16, 19, 20, 45, 50). Additionally, the statistical models employed in this study accounted for the hierarchical effects of herds nested within each site or district.

## 5 Conclusion

This study investigated the risk factors associated with LSD outbreaks in beef cattle herds in Thailand. The results revealed that herds operating for more than five years had a higher likelihood of experiencing LSD outbreaks. Additionally, herds without effective vector management practices were found to be at a greater risk of LSD outbreaks. These findings highlight the importance of implementing insect-vector control measures in LSD-risk areas, especially for herds that have not been vaccinated against LSD. This study is a significant contribution to the understanding of LSD outbreaks in Thailand. It was conducted across multiple sites. The findings can serve as guidance for managing LSD in naïve cattle herds in various settings.

## Data availability statement

The data analyzed in this study is subject to the following licenses/restrictions: the data used in this study is derived from lumpy skin disease outbreak investigations carried out by the Department of Livestock Development (DLD), Thailand, and therefore, it’s not publicly accessible. Requests to access these datasets should be directed to Department of Livestock Development (DLD), Thailand, email: dld.info@ac.th.

## Ethics statement

This study was granted ethical approval by the Walailak University Ethics Committee (WUEC-23-085-01) and was conducted in compliance with applicable guidelines and regulations. The questionnaire survey was conducted by livestock and/or veterinary authorities from the Department of Livestock Development, Ministry of Agriculture and Cooperatives. Informed consent was obtained from all subjects involved in the study.

## Author contributions

OA: Conceptualization, Data curation, Formal analysis, Investigation, Methodology, Validation, Visualization, Writing – original draft, Writing – review & editing. WW: Investigation, Validation, Writing – review & editing. BP: Investigation, Validation, Writing – review & editing. SJ: Investigation, Validation, Writing – review & editing. MS: Data curation, Investigation, Validation, Writing – original draft, Writing – review & editing. SP: Conceptualization, Data curation, Supervision, Writing – review & editing. TP: Conceptualization, Supervision, Writing – review & editing. TD: Conceptualization, Writing – review & editing. CS: Formal analysis, Validation, Visualization, Writing – review & editing. RS: Data curation, Validation, Visualization, Writing – review & editing. CJ: Data curation, Validation, Visualization, Writing – review & editing. VP: Conceptualization, Data curation, Formal analysis, Funding acquisition, Investigation, Methodology, Project administration, Resources, Software, Supervision, Validation, Visualization, Writing – original draft, Writing – review & editing.
