# Supplementary material for: Analysis of factors associated with the first lumpy skin disease outbreaks in naïve cattle herds in different regions of Thailand
Source: Front Vet Sci. 2024 Feb 22;11:1338713. doi: 10.3389/fvets.2024.1338713 (PMC10921558; doi:10.3389/fvets.2024.1338713)

Figure S1. Residual diagnostics for the final mixed-effect model, including QQ plot residuals and a plot between residuals and predicted values.

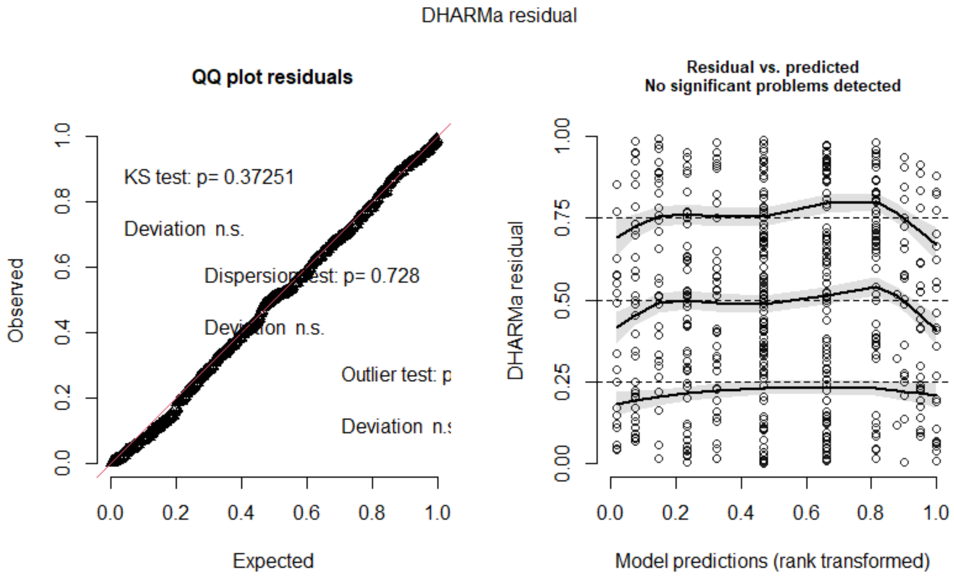

Supplement: Supplementary file 1 [file Image_1.pdf]
